# Alkyl Chain Length Effects of Imidazolium Ionic Liquids on Electrical and Mechanical Performances of Polyacrylamide/Alginate-Based Hydrogels

**DOI:** 10.3390/gels7040164

**Published:** 2021-10-05

**Authors:** Chen-Kang Chen, Po-Wen Chen, Huan-Jung Wang, Mei-Yu Yeh

**Affiliations:** 1 Department of Chemistry, Chung Yuan Christian University, No. 200, Zhongbei Rd., Zhongli Dist., Taoyuan City 320314, Taiwan; g10863026@cycu.edu.tw (C.-K.C.); g10963016@cycu.edu.tw (P.-W.C.); g10763607@cycu.edu.tw (H.-J.W.); 2 Center for Nano Technology, Chung Yuan Christian University, No. 200, Zhongbei Rd., Zhongli Dist., Taoyuan City 320314, Taiwan

**Keywords:** hydrogel, self-adhesive, polyacrylamide/alginate, imidazole, strain sensor

## Abstract

Conductive hydrogels with stretchable, flexible and wearable properties have made significant contributions in the area of modern electronics. The polyacrylamide/alginate hydrogels are one of the potential emerging materials for application in a diverse range of fields because of their high stretch and toughness. However, most researchers focus on the investigation of their mechanical and swelling behaviors, and the adhesion and effects of the ionic liquids on the conductivities of polyacrylamide/alginate hydrogels are much less explored. Herein, methacrylated lysine and different alkyl chain substituted imidazole-based monomers (IMCx, x = 2, 4, 6 and 8) were introduced to prepare a series of novel pAMAL-IMCx-Ca hydrogels. We systematically investigated their macroscopic and microscopic properties through tensile tests, electrochemical impedance spectra and scanning electron microscopy, as well as Fourier transform infrared spectroscopy, and demonstrated that an alkyl chain length of the IMCx plays an important role in the designing of hydrogel strain sensors. The experiment result shows that the hexyl chains of IMC6 can effectively entangle with LysMA through hydrophobic and electrostatic interactions, which significantly enhance the mechanical strength of the hydrogels. Furthermore, the different strain rates and the durability of the pAMAL-IMC6-Ca hydrogel were investigated and the relative resistance responses remain almost the same in both conditions, making it a potential candidate for wearable strain sensors.

## 1. Introduction

Stretchable, flexible, wearable and environmentally friendly electronic devices are of great significance to meet the increasing demands for versatility and complexity of modern electronics [1,2,3,4,5,6,7,8]. The strain sensors have attracted tremendous attention recently since they can convert mechanical deformations into electrical signals, and they are widely used in personalized healthcare [9,10], human-machine interaction [11,12,13], human motion detection [14,15], physiological monitoring [16,17], electronic skins [18,19,20,21,22], artificial intelligence [23] and so on. Most conventional strain sensors fabricated by metals, semiconductors and carbon-based materials only can sense small deformations of less than 5% strain [24] and have poor recoverability, making them uncomfortable to wear and difficult to get used to. A hydrogel strain sensor provides a feasible solution for wearable devices, not only because of its super extensibility and high flexibility, but also because of its good biocompatibility [25,26,27,28]. 

The polyacrylamide/alginate-based hydrogels have been studied by many researchers because of their tunable mechanical properties and biocompatibility [29,30,31,32,33]. For example, Suo and co-workers established the first example of double-network hydrogel with high stretch and toughness based on polyacrylamide/Ca-alginate, thus opening new application areas for this class of materials [29]. Zhou and Chen discovered that the mechanical strength of polyacrylamide/alginate-based hydrogel can be tuned by immersing it in an aqueous solution of CaCl_2_, SrCl_2_, BaCl_2_, AlCl_3_ or Fe(NO_3_)_3_ [30]. Kim et al. designed fabrication of super-stiff, anisotropic hybrid hydrogels through linear remodeling of polyacrylamide/Ca-alginate hydrogel and followed by a secondary crosslinking with various cations (Ba, Al or Fe ions) [31]. Suo and Vlassak combined short- and long-chain alginates to reduce the viscosity of pregel solutions and successfully demonstrated that polyacrylamide/alginate hydrogels can simultaneously have high stiffness and toughness [32]. Nonetheless, many research efforts are focused on the investigation of the mechanical behaviors and/or the swelling characteristics, the adhesion properties [34,35], and the effects of the ionic liquids on the conductivities of polyacrylamide/alginate-based hydrogels are much less explored.

In this work, we developed a series of novel self-adhesive pAMAL-IMCx-Ca hydrogels based on polyacrylamide/Ca-alginate hydrogels (x = 2, 4, 6 and 8). Herein, the methacrylated lysine (LysMA) was used to provide the self-adhesive property of pAMAL-IMCx-Ca hydrogels because LysMA has superior adhesion for many substrates [36]. Furthermore, imidazole is an important heterocyclic structural motif in functional molecules, whose derivatives are widely used as ionic liquids, drugs, catalysts and dyes [37,38]. Therefore, the imidazolium ionic liquid monomers (IMCx) with various chain lengths were incorporated to fine tune the electrical and mechanical performances of the hydrogels. From the electron microscopic study, FTIR, mechanical and electrochemical investigations of pAMAL-IMCx-Ca hydrogels, we demonstrated that pAMAL-IMC6-Ca displayed the optimal performance, which could be attributed to the balance between electrostatic interaction, metal-ligand coordination and hydrogen bonding interaction, as well as hydrophobic interaction. In the presence of the good mechanical properties, conductivity, adhesive performance and sensitivity, the pAMAL-IMC6-Ca hydrogel exhibited potential applications in the field of wearable strain sensors.

## 2. Results and Discussion

### 2.1. Design Strategy and Characterization of the pAMAL-IMCx-Ca Hydrogels

**Figure 1:**
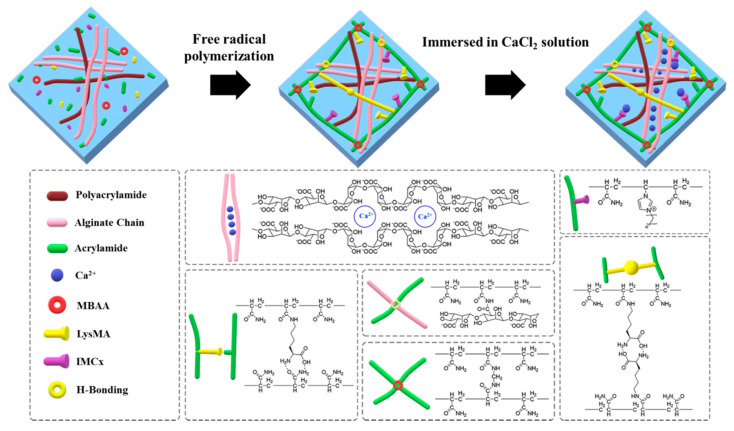
A schematic image of the fabrication process and formation mechanism of pAMAL-IMCx-Ca hydrogels (x = 2, 4, 6 and 8).

For use in wearable sensor applications, the hydrogels should exhibit mechanical strength, and stretchable, flexible, conductivity and adhesive properties. We used acrylamide, *N*,*N′*-methylenebisacrylamide (MBAA), alginate and LysMA to prepare adhesive hydrogels (defined as AMAL). However, AMAL showed poor formability, and polyacrylamide was utilized in order further to improve the toughness of gel, which is called pAMAL. For the purpose of enhancing the elasticity and conducting electricity of the hydrogels, CaCl_2_ and IMCx were added. Herein, we chose the CaCl_2_, since it is known that alginate can interact with the Ca ion through the metal-ligand coordination and effectively improve its mechanical properties [29,32,33]. Additionally, there may exist electrostatic interaction between the imidazole cations in IMCx and the carboxylate groups in alginate and/or LysMA [39]. Furthermore, the alkyl chain length of the imidazolium ionic liquid could also be used to explore the electrical conductivities of the hydrogels [40]. Figure 1 shows the fabrication process and the suppositional mechanism of the newly discovered pAMAL-IMCx-Ca hydrogels (x = 2, 4, 6 and 8). Acrylamide, MBAA, LysMA and IMCx will form a covalent bond by one-pot radical polymerization. Aqueous solutions of the above polymers and Ca-alginate formed a double-network hydrogel, which may be achieved by entanglements of the polymers to obtain stretchable hydrogels [29,33,41,42]. Moreover, the metal-ligand coordination, electrostatic interaction and hydrogen bonding interaction could provide additional stability to the pAMAL-IMCx-Ca hydrogels.

**Figure 2:**
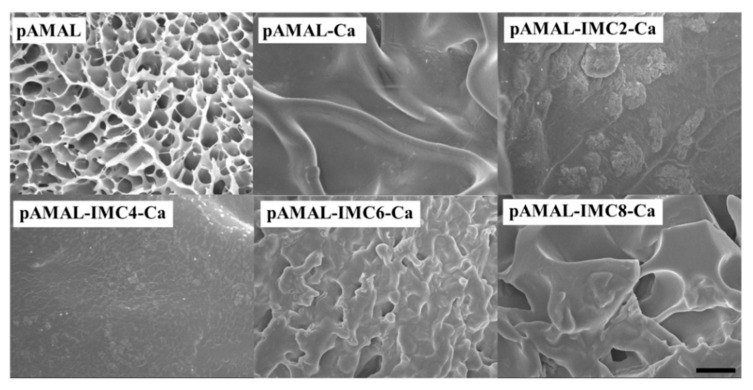
SEM images of pAMAL, pAMAL-Ca, pAMAL-IMC2-Ca, pAMAL-IMC4-Ca, pAMAL-IMC6-Ca and pAMAL-IMC8-Ca hydrogels. Scale bar: 10 μm.

A series of IMCx of 1-vinyl-3-ethylimidazolium bromide (IMC2), 1-vinyl-3-butylimidazolium bromide (IMC4), 1-vinyl-3-hexylimidazolium bromide (IMC6) and 1-vinyl-3-octylimidazolium bromide (IMC8) was synthesized to investigate the alkyl chain length effect on the mechanical and electrical performances of the pAMAL-based hydrogels. The hydrogels of pAMAL, pAMAL-IMC2, pAMAL-IMC4, pAMAL-IMC6 and pAMAL-IMC8 were immersed in CaCl_2_ aqueous solution to obtain the corresponding pAMAL-Ca, pAMAL-IMC2-Ca, pAMAL-IMC4-Ca, pAMAL-IMC6-Ca and pAMAL-IMC8-Ca, respectively (Table S1). We found that pAMAL was a transparent hydrogel, and the appearance of the hydrogels was opaque when CaCl_2_ was incorporated (Figure S1). It is interesting to note that pAMAL-IMC2-Ca was a soft and weak gel, pAMAL-IMC4-Ca was a soft and highly stretchable gel, pAMAL-IMC6-Ca was a stretchable and strong gel and pAMAL-IMC8-Ca was a stable gel. These observations were probably due to strong hydrophobic interaction between IMCx and the balance of the hydrophobicity and hydrophilicity of the water environment. The microstructures of these hydrogels were examined by scanning electron microscopy (SEM). As can be seen from Figure 2, the surface of the pAMAL hydrogel was clearly observed to be a typical three-dimensional porous network, and the pore size was within the range of 3–10 μm. As the Ca ion and IMCx were introduced into the pAMAL gels, the porous network was covered in varying degrees, depending on the type of IMCx. The pAMAL-Ca hydrogel showed continuously connected structure, suggesting that it may have larger mechanical strength than that of the pAMAL hydrogel. It is interesting to note that the hydrogel morphology can change dramatically upon introducing the IMCx species. The lysine molecule possesses both a carboxyl group and a nonpolar part (butyl chain with relatively hydrophobic feature) [43], which is available for interaction with IMC6. The cooperative effect of the hydrophobic interaction between the butyl chain of LysMA and the hexyl chain of IMC6 as well as the electrostatic force between carboxylate group and imidazole cation participate in forming the hybrid hydrogel. As a result, multilayer microstructure was observed in the pAMAL-IMC6-Ca sample, which indicated that the pAMAL-IMC6-Ca hydrogel had the most elasticity among the six hydrogels. IMC2 and IMC4 do not provide enough hydrophobic driving force to entangle with LysMA, and thus the relative smooth morphologies of pAMAL-IMC2-Ca and pAMAL-IMC4-Ca hydrogels can be observed. On the contrary, IMC8 has a longer hydrophobic segment that decreases its solubility in the aqueous solution [44], and we found the block and heterogeneous morphology in the pAMAL-IMC8-Ca sample under SEM microscopy. In fact, the cloudy solution can be seen in the preparation process of the pAMAL-IMC8-Ca hydrogel with the naked eye.

**Figure 3:**
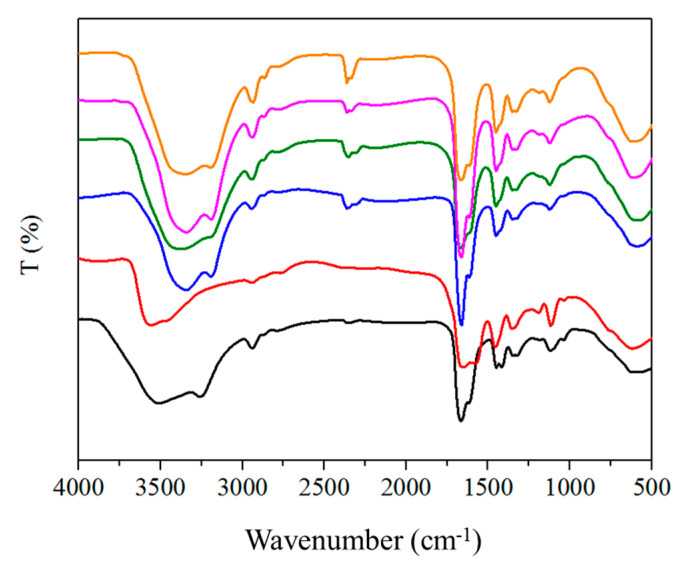
FTIR spectra of pAMAL-based hydrogels (pAMAL for black; pAMAL-Ca for red; pAMAL-IMC2-Ca for blue; pAMAL-IMC4-Ca for olive; pAMAL-IMC6-Ca for magenta; pAMAL-IMC8-Ca for orange).

To prove our speculation of the proposed interaction mechanism between pAMAL, the Ca ion and IMCx, Fourier transform infrared spectroscopy (FTIR) was carried out to characterize the composite hydrogels. From the FTIR spectrum of pAMAL, the broad absorption peaks around 3500 cm^−^^1^ can be attributed to the stretching vibration of OH and NH groups [45]. The absorption bands at 1680 and 1615 cm^−^^1^ were assigned to C=O stretching of carboxylic acid and amide, respectively [46]. Moreover, the peak at 1119 cm^−^^1^ was caused by the C–O stretching vibration [47]. These characteristic bands are confirmed the existence of alginate, lysine residue and (poly)acrylamide in pAMAL gels. As shown in Figure 3, the C=O peaks were shifted to lower wavenumbers from 1680 to 1657 cm^−^^1^ and 1615 to 1573 cm^−^^1^ with the addition of CaCl_2_, respectively, suggesting the formation of the metal-ligand coordination in pAMAL-Ca hydrogel [48]. Moreover, the absorption bands owing to OH and NH groups shifted to lower wavenumbers and appeared at ca. 3350 cm^−^^1^ in the cases of adding IMCx, demonstrating the existence of the intermolecular hydrogen bonding interaction in pAMAL-IMCx-Ca systems (x = 2, 4, 6 and 8) [49,50].

### 2.2. Mechanical Properties

**Figure 4:**
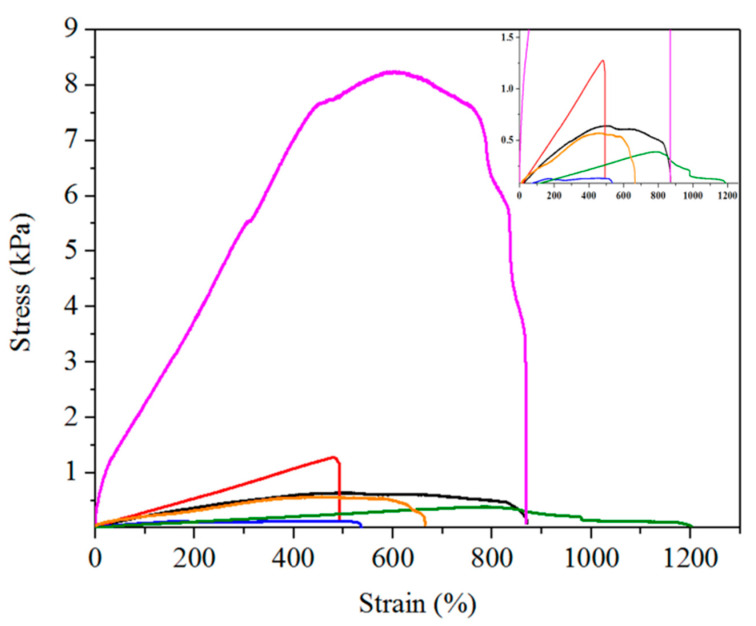
The tensile stress–strain curves of pAMAL-based hydrogels and inset are the partial enlarged details (pAMAL for black; pAMAL-Ca for red; pAMAL-IMC2-Ca for blue; pAMAL-IMC4-Ca for olive; pAMAL-IMC6-Ca for magenta; pAMAL-IMC8-Ca for orange).

The key to the practical application of hydrogels for wearable strain sensors is good mechanical property [51,52,53]. We carried out tensile tests to study the mechanical properties of pAMAL-based hydrogels. Figure 4 shows the stress–strain curves of pAMAL, pAMAL-Ca, pAMAL-IMC2-Ca, pAMAL-IMC4-Ca, pAMAL-IMC6-Ca and pAMAL-IMC8-Ca hydrogels. We observed that when pAMAL hydrogel was immersed into the Ca ion solution, the tensile strength of pAMAL-Ca hydrogel increased by 200% compared to that of the pAMAL hydrogel, while the elongation at break of pAMAL-Ca was reduced by 56% (Table 1). In order to enhance the elongation at break of pAMAL-Ca hydrogel, IMCx were introduced. We systematically investigated the effect of the hydrophobic alkyl monomers (IMC2, IMC4, IMC6 and IMC8) on the mechanical properties of the pAMAL-Ca hydrogel (Figure 4 and Figure S2, Table 1). The hydrogels of pAMAL-IMC2-Ca and pAMAL-IMC4-Ca exhibited larger elongation at break compared with pAMAL-Ca hydrogel, which can be attributed to the electrostatic interaction between imidazole cations and carboxylate in alginate and/or LysMA [39]. However, the pAMAL-IMC2-Ca and pAMAL-IMC4-Ca hydrogels displayed lower tensile strength than that of the pAMAL-Ca hydrogel. This result could be explained by the competition of imidazole cations and Ca ions for carboxylate group, leading to the decrease of metal-ligand coordination and the increase of electrostatic interaction [39,54]. Interestingly, the pAMAL-IMC6-Ca hydrogel showed optimal mechanical properties, with an improvement of 645% in tensile strength, 177% in elongation at break and 617% in Young’s modulus, compared to the pAMAL-Ca hydrogel. The significant enhancement of mechanical strength of the pAMAL-IMC6-Ca hydrogel could be due to the hydrophobic interactions of long alkyl chains on imidazole cations [55]. It is worth noting that the mechanical performance of the pAMAL-IMC8-Ca hydrogel is much lower than that of the pAMAL-IMC6-Ca hydrogel, probably owing to IMC8 with very long alkyl chains (i.e., too hydrophobic) which decrease solubility in water [44]. Additionally, the swelling test was carried out to better understand the degree of crosslinking of the pAMAL-based hydrogels, i.e., the higher the crosslink density, the lower the swelling ratio [56,57]. We found that the pAMAL-IMC6-Ca hydrogel displayed lower swelling ratio than that of the pAMAL-Ca (51% and 364%, respectively), while the other 4 hydrogels collapsed after being soaked in water for 5 days. These findings are consistent with the result of the mechanical strength of pAMAL-based hydrogels. 

### 2.3. Electrical Properties 

**Figure 5:**
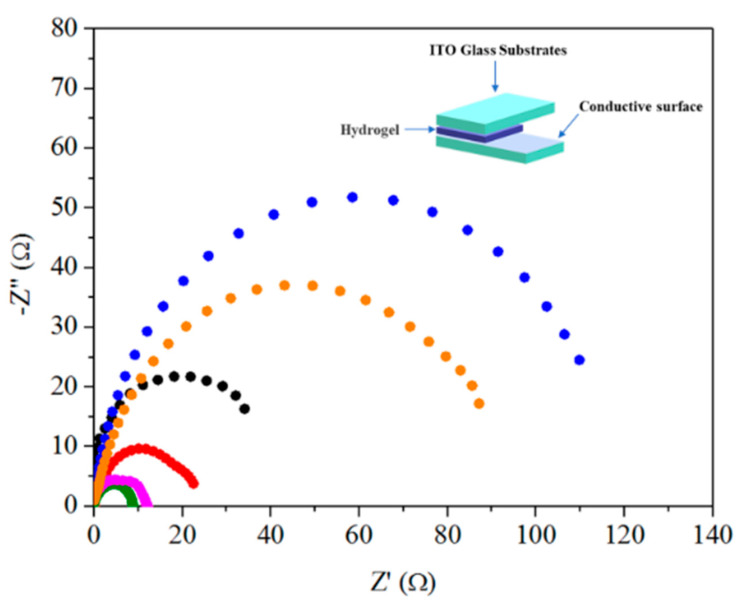
Nyquist impedance spectra of pAMAL-based hydrogels (pAMAL for black; pAMAL-Ca for red; pAMAL-IMC2-Ca for blue; pAMAL-IMC4-Ca for olive; pAMAL-IMC6-Ca for magenta; pAMAL-IMC8-Ca for orange). Inset is the schematic diagram of the electrochemical test device.

The electrical conductivity of hydrogel is one of the most important properties of hydrogels used in wearable devices [58,59]. The electrochemical impedance spectra (EIS) of the pAMAL-based hydrogels were measured by an electrochemical workstation. The results of resistance, resistivity and conductivity for the six samples are presented in Figure 5 and Table 1. The resistance of the pAMAL and pAMAL-Ca hydrogels was 42.0 and 24.1 Ω, respectively. That is because CaCl_2_ possesses good conductivity and consequently reduces the resistance of pAMAL-Ca [60]. Moreover, the pAMAL-Ca hydrogel showed a higher swelling ratio than that of the pAMAL-IMC6-Ca sample, and it can be speculated that the pAMAL-Ca gel has a better Ca ion adsorption capacity. The resistance of the pAMAL-Ca and pAMAL-IMC6-Ca was 24.1 and 11.2 Ω, respectively, implying that the ionic liquid is the dominant factor that contributes to the hydrogel conductivity. The effect of the alkyl chain length of imidazole cations, i.e., IMC2, IMC4, IMC6 and IMC8, on the electrical properties was also investigated. Unexpectedly, the pAMAL-IMC2-Ca hydrogel showed high electrical resistance among these hydrogels, which could be due to the poor mechanical properties and bad formability of pAMAL-IMC2-Ca gel (Figure 4 and Figure S1, Table 1). In addition, the electrical resistance increased with the increase of the alkyl chain length of IMCx, and the resistance of the pAMAL-IMC4-Ca, pAMAL-IMC6-Ca and pAMAL-IMC8-Ca hydrogels was 8.3, 11.2 and 97.5 Ω, respectively. As indicated in Figure 5, the resistance (and resistivity) of the hydrogels is on the order of pAMAL-IMC4-Ca < pAMAL-IMC6-Ca < pAMAL-Ca < pAMAL < pAMAL-IMC8-Ca < pAMAL-IMC2-Ca. Since resistivity is the opposite of conductivity [61], the pAMAL-IMC4-Ca and pAMAL-IMC6-Ca hydrogels had better conductivity than the other hydrogels. Based on the SEM, FTIR, mechanical and electrochemical properties demonstrated above, the pAMAL-IMC6-Ca hydrogel was found to exhibit the optimal performance, which could be attributed to the balance between electrostatic interaction, metal-ligand coordination, hydrogen bonding interaction and hydrophobic interaction.

### 2.4. Adhesive Capacity and Strain Sensor Testing

**Figure 6:**
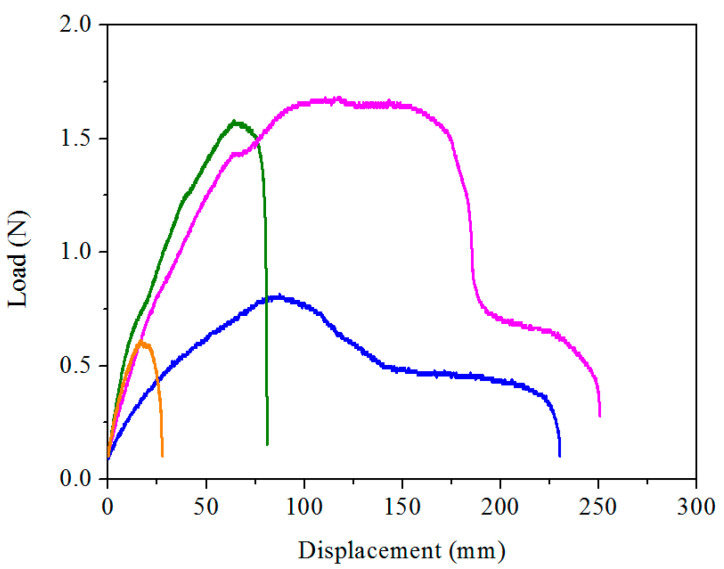
Shear strength tests of pAMAL-based hydrogels (pAMAL-IMC2-Ca for blue; pAMAL-IMC4-Ca for olive; pAMAL-IMC6-Ca for magenta; pAMAL-IMC8-Ca for orange).

**Figure 7:**
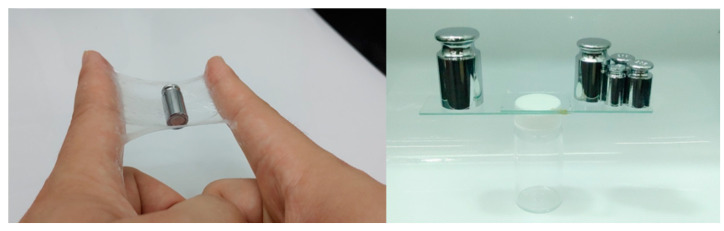
The pAMAL-IMC6-Ca hydrogel exhibited good adhesiveness and load-bearing properties.

**Figure 8:**
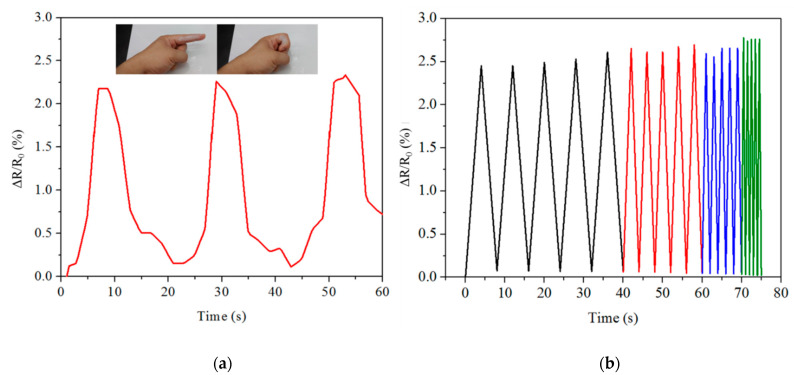
(**a**) Relative resistance changes for the stretching and bending of the index finger (pAMAL-IMC6-Ca hydrogel). (**b**) Relative resistance change under different stretch frequencies (0.1 Hz for black; 0.5 Hz for red; 1.0 Hz for blue; 2.0 Hz for olive).

Except for the mechanical strength and conductivity of hydrogels, good adhesive capacity is vital for wearable electronics applications [62,63,64]. In order to evaluate the adhesion abilities of pAMAL-IMCx-Ca hydrogels, we measured the shear strength of the hydrogels by lap-shear tests [65,66]. From the data reported in Figure 6, the pAMAL-IMC6-Ca sample was found to be more adhesive than other samples (shear strength ca. 4.2 kPa). As revealed in Figure S3, pAMAL-IMC6-Ca hydrogel showed strong adhesion to numerous materials, such as wood, plastic, glass and rubber, suggesting a substantial broad application in various substrates of the pAMAL-IMC6-Ca hydrogel. Furthermore, the adhesiveness of the pAMAL-IMC6-Ca hydrogel is strong enough to adhere to gloves and capable of stretching up to 500% of its original length (Figure S3). As can be seen from Figure 7, the pAMAL-IMC6-Ca hydrogel placed between the two fingers or glass slides could support weights up to 200 g without adhesives. In addition to a load-bearing test, we also attempted to shake fingers back and forth quickly to examine the adhesive capacity of the pAMAL-IMC6-Ca hydrogel (Video S1 and Video S2). These results all suggest that the pAMAL-IMC6-Ca hydrogel exhibited good adhesiveness and load-bearing properties. Due to its excellent adhesion, it can be attached to the dynamic movement parts of the human body to directly monitor the changes of the signal. Figure 8a shows that the pAMAL-IMC6-Ca hydrogel responds quickly and repeatedly to the movement of the index finger when it is bent or stretched. Moreover, the different strain rates (0.1–2.0 Hz) and the durability of the pAMAL-IMC6-Ca hydrogel were also conducted (Figure 8b and Figure S4). The relative resistance change of the pAMAL-IMC6-Ca strain sensor was nearly independent of the frequency within the tested frequency range, implying the excellent stability of the sensor under diverse strain rates. In addition, after 1000 stretch/release cycles, the relative resistance response still remains almost the same, making it a potential candidate for wearable strain sensors.

## 3. Conclusions

A series of novel pAMAL-IMCx-Ca hydrogels with tunable mechanical strengths and conductivities were fabricated (x = 2, 4, 6 and 8). We systematically investigated their macroscopic and microscopic properties through mechanical tensile stress–strain tests, EIS, SEM and FTIR, and demonstrated that the alkyl chain length effects of the IMCx play an important role in the designing of hydrogel strain sensors. The experiment result shows that the hexyl chains of IMC6 can effectively entangle with LysMA through hydrophobic and electrostatic interactions, which significantly enhance the mechanical strength of the hydrogels. Furthermore, the different strain rates and the durability of the pAMAL-IMC6-Ca hydrogel were investigated, and we found that the relative resistance responses remain almost the same in both conditions, making it a potential candidate for wearable strain sensors.

## 4. Materials and Methods

### 4.1. Materials

Acrylamide, polyacrylamide (average MW 10,000) and trifluoroacetic acid were obtained from Acros Organics (Geel, Antwerp, Belgium). Potassium persulfate, sodium alginate and methacrylic anhydride were purchased from Sigma-Aldrich (St. Louis, MO, USA). *N*,*N′*-Methylenebisacrylamide, 1-vinylimidazole, bromoethane, 1-bromobutane, 1-bromohexane and 1-bromooctane were gained from Alfa Aesar (Ward Hill, MA, USA). Calcium chloride and Boc-Lys-OH were obtained from Merck (Darmstadt, Germany) and Carbosynth (Compton, UK), respectively. Deionized water was used in all the experiments.

### 4.2. Synthesis of Monomers

Methacrylated lysine (LysMA), 1-vinyl-3-ethylimidazolium bromide (IMC2), 1-vinyl-3-butylimidazolium bromide (IMC4), 1-vinyl-3-hexylimidazolium bromide (IMC6) as well as 1-vinyl-3-octylimidazolium bromide (IMC8) were synthesized according to literature procedures with suitable modifications [36,67]. The chemical structures of LysMA, IMC2, IMC4, IMC6 and IMC8 were characterized by ^1^H NMR spectra (see Supplementary Materials for detailed analyses).

Synthesis of LysMA: Boc-Lys-OH (0.50 g, 2.0 mmol) and NaHCO_3_ (0.34 g, 4.1 mmol) were dissolved in a mixed solution of THF/deionized water (7 mL, *v*/*v* = 6/1). Methacrylic anhydride (3.95 mL) in 7.5 mL THF was added dropwise into above reaction mixture over 15 min at 0 °C and then warmed to room temperature. After reaction for 24 h, THF was removed by rotary evaporation and the pH of the sample was adjusted to 2.0 using 0.1 M HCl aqueous solution. The sample was then extracted with CH_2_Cl_2_ and dried to give yellow oil-like crude product (Boc-LysMA-OH). The trifluoroacetic acid (15 mL) and CH_2_Cl_2_ (15 mL) were added to the crude product of Boc-LysMA-OH, and the reaction mixture was stirred at room temperature for 2 h. Subsequently, the reaction solution was poured into the mixture of hexane (15 mL) and ether (15 mL) and stored in refrigerator at −20 °C for 6 h. The white viscous solid was collected and dissolved in ethanol, followed by adding triethylamine to obtain a white precipitate of LysMA in 57% yield.

Synthesis of IMC2, IMC4, IMC6 and IMC8: Bromoethane (4.63 g, 42.5 mmol) was added to 1-vinylimidazole (1.00 g, 10.6 mmol) and the reaction mixture was heated to 70 °C for 24 h. After cooling to room temperature, the upper phase was poured out and the remaining liquid residue was washed with ethyl acetate (EA, 30 mL) for 3 times. EA was removed by rotary evaporation at 60 °C for 2 h to obtain a transparent liquid of IMC2 (yield: 78%). In a manner similar to that described above, IMC4, IMC6 and IMC8 were synthesized by reaction of 1-bromobutane (5.82 g, 42.5 mmol) with 1-vinylimidazole (1.00 g, 10.6 mmol), 1-bromohexane (7.57 g, 42.5 mmol) with 1-vinylimidazole (1.00 g, 10.6 mmol) as well as 1-bromooctane (8.21 g, 42.5 mmol) with 1-vinylimidazole (1.00 g, 10.6 mmol), respectively (yields: 74–78%).

### 4.3. Preparation of pAMAL-Based Hydrogels

Preparation of pAMAL hydrogel: Acrylamide (400 mg), sodium alginate (50 mg) and LysMA (30 mg) were dissolved in 2.5 mL of deionized water to gain a transparent solution. Thereafter, 500 μL of potassium persulfate solution (0.02 g/mL), 500 μL of polyacrylamide solution (1 mg/mL) and 100 μL of *N*,*N′*-methylenebisacrylamide solution (1 mg/mL) were added to obtain homogeneous solution. Subsequently, the above homogeneous solution was heated to 70 °C for 6 h to undergo the polymerization reaction and the resulting hydrogel was defined as pAMAL hydrogel.

Preparation of pAMAL-Ca hydrogel: Acrylamide (400 mg), sodium alginate (50 mg) and LysMA (30 mg) were dissolved in 2.5 mL of deionized water to gain a transparent solution. Thereafter, 500 μL of potassium persulfate solution (0.02 g/mL), 500 μL of polyacrylamide solution (1 mg/mL) and 100 μL of *N*,*N′*-methylenebisacrylamide solution (1 mg/mL) were added to obtain homogeneous solution. Subsequently, the above homogeneous solution was heated to 70 °C for 6 h. After 6 h, the hydrogel was formed and cooled to room temperature, then soaked in an aqueous solution consisting of 0.4 M of CaCl_2_ for 6 h. The resulting hydrogel was defined as pAMAL-Ca hydrogel. 

Preparation of pAMAL-IMCx-Ca hydrogel: Acrylamide (400 mg), sodium alginate (50 mg) and LysMA (30 mg) were dissolved in 2.5 mL of deionized water to gain a transparent solution. Thereafter, 500 μL of potassium persulfate solution (0.02 g/mL), 500 μL of polyacrylamide solution (1 mg/mL), 100 μL of *N*,*N′*-methylenebisacrylamide solution (1 mg/mL) as well as 30 μL IMC2 were added to the above transparent solution and the mixture was heated to 70 °C for 6 h. After 6 h, the hydrogel was formed and cooled to room temperature, then soaked in an aqueous solution consisting of 0.4 M of CaCl_2_ for 6 h. The resulting hydrogel was defined as pAMAL-IMC2-Ca hydrogel. The pAMAL-IMC4-Ca, pAMAL-IMC6-Ca and pAMAL-IMC8-Ca hydrogels were prepared by replacing IMC2 with IMC4, IMC6 and IMC8, respectively. 

### 4.4. Measurements

Nuclear magnetic resonance (NMR) spectra were conducted by Bruker (Billerica, MA, USA) AVANCEII-400 MHz spectrometer with D_2_O as the solvent. The morphologies of hydrogels were observed using the scanning electron microscope (SEM, JSM-7600F) and the molecular interactions of the hydrogels were characterized using a Thermo Fisher Scientific (Waltham, MA, USA) Nicolet iS5 Fourier transform infrared (FTIR) spectrometer. Both of the test samples were prepared via drying the hydrogels by freeze vacuum drier. For SEM samples preparation, the hydrogel samples were rapidly frozen in liquid nitrogen and further lyophilized with a freeze dryer system under vacuum at −80 °C for at least 24 h until all of the water was sublimed. Freeze-dried hydrogel samples were coated with platinum under vacuum, and were used to investigate the morphology of the pAMAL-based hydrogels utilizing a Jeol (Tokyo, Japan) JSM-7600F scanning electron microscopy [68,69]. The mechanical tests were performed on a tensile tester (Gotech AI-3000-U, Taichung, Taiwan). The lap-shear test was conducted to examine the adhesion strength of the hydrogels (with a load cell 10 N at a rate of 5 mm/min). The lap shear strength was calculated using Equation (1):

Lap shear strength (Pa) = maximum loading force (N)/bonging area (m^2^) (1)

The electrochemical measurements were recorded with CH Instruments (Austin, TX, USA) CHI627E Electrochemical Workstation. The electrochemical cell with a sandwich structure was fabricated by assembling the conductive hydrogel sheet in the middle of two indium tin oxide (ITO) glass substrates, wherein the thickness of the conductive hydrogel was 0.08 mm. The conductivity (σ, S/cm) was calculated according to the Equation (2):

σ = L/(R × A)(2)

where L was the length between ITO glass substrates, R and A represented the resistance and the cross-sectional area of hydrogel samples, respectively.

The relative changes of hydrogel resistance were calculated by the Equation (3):

ΔR/R_0_ = (R − R_0_)/R_0_ × 100% (3)

where R_0_ and R were the resistance of hydrogel without and with the imposed strain, respectively.

## Figures and Tables

**Table 1:** Mechanical and electrical properties of pAMAL-based hydrogels.

Hydrogels	Tensile Strength (kPa)	Elongation at Break (%)	Young’s Modulus (kPa)	Rs (Ω)	Resistivity (kΩ·cm)	Conductivity (μS/cm)
pAMAL	0.64	871	0.17	42.0	5.3	190.5
pAMAL-Ca	1.28	491	0.24	24.1	3.0	332.0
pAMAL-IMC2-Ca	0.13	537	0.09	120.2	15.0	66.6
pAMAL-IMC4-Ca	0.39	1201	0.04	8.3	1.0	969.7
pAMAL-IMC6-Ca	8.25	868	1.48	11.2	1.4	714.3
pAMAL-IMC8-Ca	0.57	665	0.11	97.5	12.2	82.1

## Supplementary Materials

The following are available online at https://www.mdpi.com/article/10.3390/gels7040164/s1, Table S1: The composition of the pAMAL-based hydrogels, Figure S1: The photographs of pAMAL, pAMAL-Ca, pAMAL-IMC2-Ca, pAMAL-IMC4-Ca, pAMAL-IMC6-Ca and pAMAL-IMC8-Ca hydrogels, Figure S2: The tensile strengths of pAMAL-based hydrogels. Error bars show standard deviation, Figure S3: Exhibition of adhesion between pAMAL-IMC6-Ca hydrogel and various substrates (wood, plastic, glass and rubber), Figure S4: Relative resistance change over 1000 cycles (pAMAL-IMC6-Ca hydrogel), Figure S5: The proton NMR spectrum of Lys-MA in D_2_O, Figure S6: The proton NMR spectrum of IMC2 in D_2_O, Figure S7: The proton NMR spectrum of IMC4 in D_2_O, Figure S8: The proton NMR spectrum of IMC6 in D_2_O, Figure S9: The proton NMR spectrum of IMC8 in D_2_O, Video S1: Adhesive testing of pAMAL-IMC6-Ca hydrogel (finger), Video S2: Adhesive testing of pAMAL-IMC6-Ca hydrogel (gloves).
