# Supplementary material for: Alkyl Chain Length Effects of Imidazolium Ionic Liquids on Electrical and Mechanical Performances of Polyacrylamide/Alginate-Based Hydrogels
Source: Gels. 2021 Oct 5;7(4):164. doi: 10.3390/gels7040164 (PMC8544473; doi:10.3390/gels7040164)
Supplement: Supplementary file 1 [file gels-07-00164-s001.zip › gels-1387323-supplementary.pdf]

gels

Alkyl Chain Length Effects of Imidazolium Ionic Liquids on Electrical and Mechanical Performances of Polyacrylamide/alginate-based Hydrogels

Chen-Kang Chen [1], Po-Wen Chen [1], Huan-Jung Wang [1] and Mei-Yu Yeh [1,2,*]

[1] Department of Chemistry, Chung Yuan Christian University, 200, Zhongbei Rd., Zhongli, Taiwan; g10863026@cycu.edu.tw (C.-K. Chen); g10963016@cycu.edu.tw (P.-W. Chen); g10763607@cycu.edu.tw (H.-J. Wang)
[2] Center for Nano Technology, Chung Yuan Christian University
* Correspondence: myyeh@cycu.edu.tw; Tel.: +886-3-265-3335

Supplementary Information (SI)

Table S1: The composition of the pAMAL-based hydrogels[a]

Hydrogels	Acrylamide[c]	MBAA[c]	Alginate[c]	polyacrylamide[c]	LysMA[c]	IMCx[c,d]
pAMAL	400	0.1	50	0.5	30	0
pAMAL-Ca[b]	400	0.1	50	0.5	30	0
pAMAL-IMC2-Ca[b]	400	0.1	50	0.5	30	30
pAMAL-IMC4-Ca[b]	400	0.1	50	0.5	30	34.5
pAMAL-IMC6-Ca[b]	400	0.1	50	0.5	30	38.8
pAMAL-IMC8-Ca[b]	400	0.1	50	0.5	30	43.1

[a] Potassium persulfate 0.1% w/v, 2.5 mL deionized water. [b] 0.4 M of $CaCl_2$ aqueous solution. [c] Unit: mg. [d] IMCx was 0.15 mmol. (x=2, 4, 6, 8)

Figure S1: The photographs of pAMAL, pAMAL-Ca, pAMAL-IMC2-Ca, pAMAL-IMC4-Ca, pAMAL-IMC6-Ca and pAMAL-IMC8-Ca hydrogels.

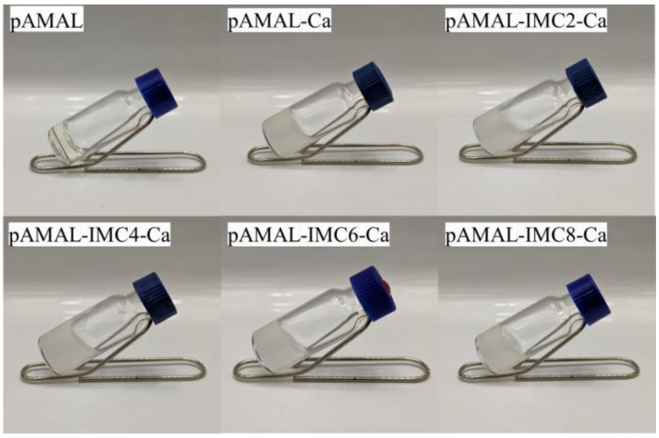

Figure S2: The tensile strengths of pAMAL-based hydrogels. Error bars show standard deviation.

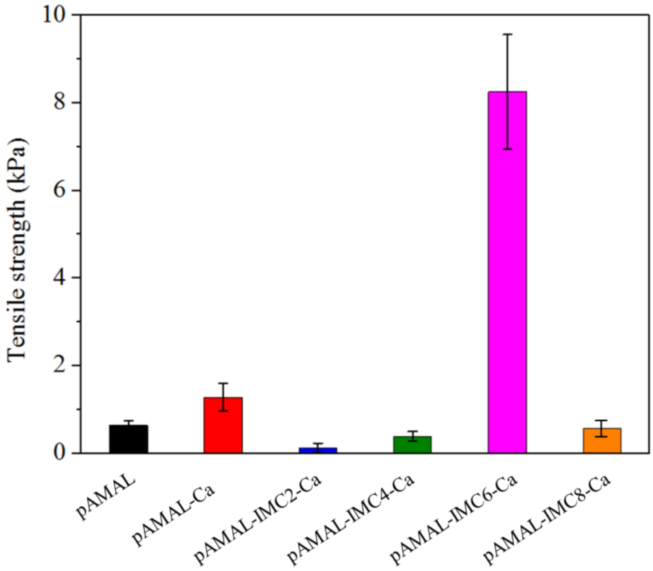

Figure S3: Exhibition of adhesion between pAMAL-IMC6-Ca hydrogel and various substrates (wood, plastic, glass and rubber).

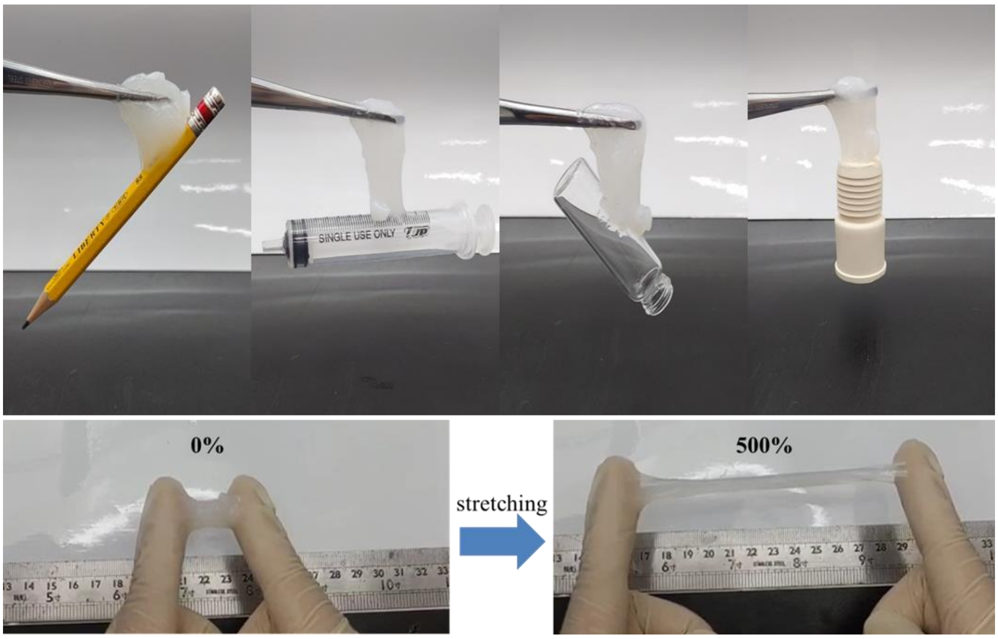

Figure S4: Relative resistance change over 1000 cycles (pAMAL-IMC6-Ca hydrogel).

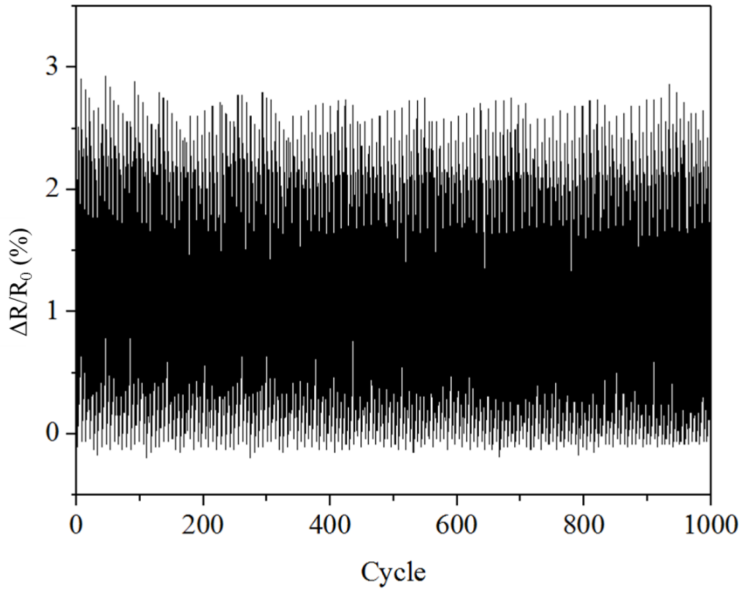

Figure S5: The proton NMR spectrum of Lys-MA in D_2O.

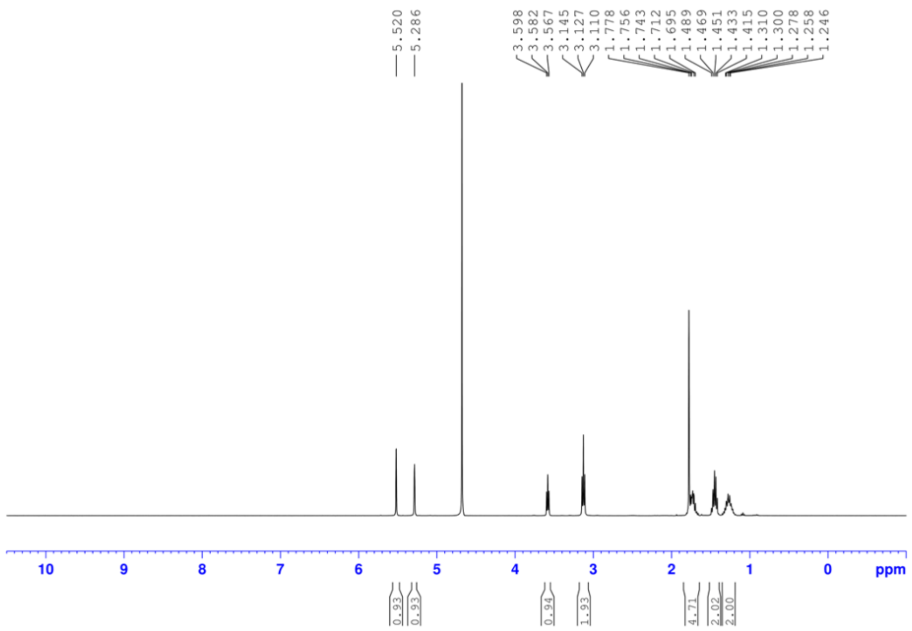

Figure S6: The proton NMR spectrum of IMC2 in D_2O.

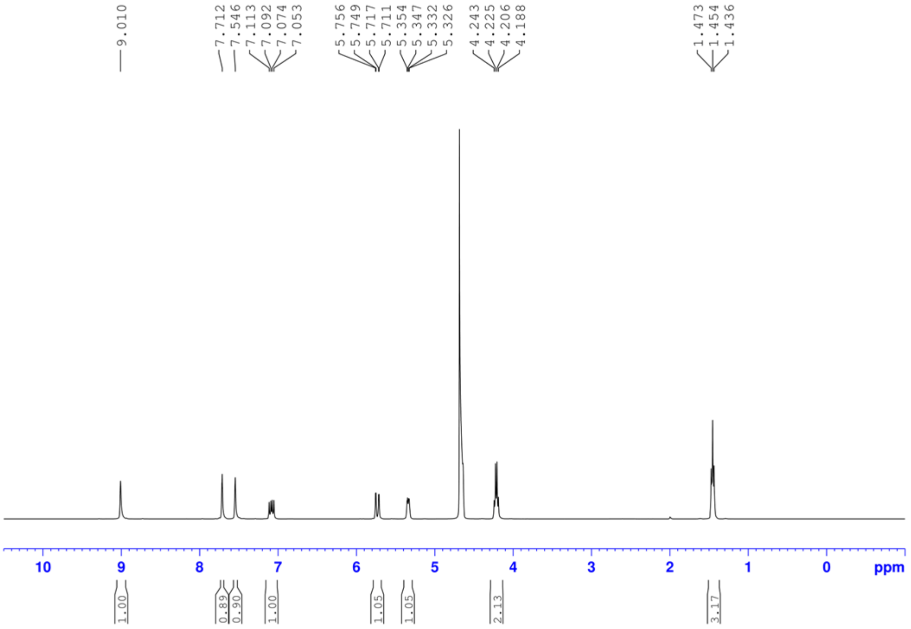

Figure S7: The proton NMR spectrum of IMC4 in D_2O.

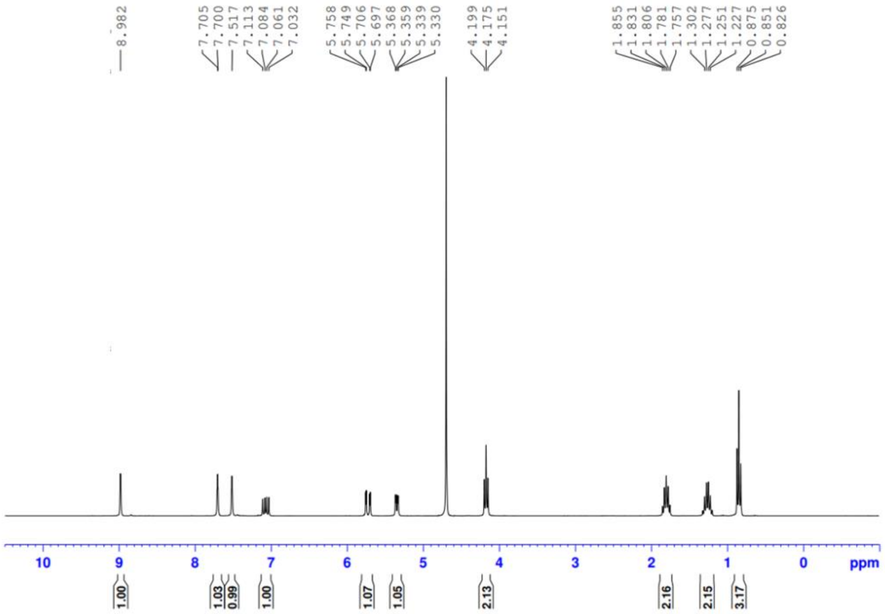

Figure S8: The proton NMR spectrum of IMC6 in D_2O.

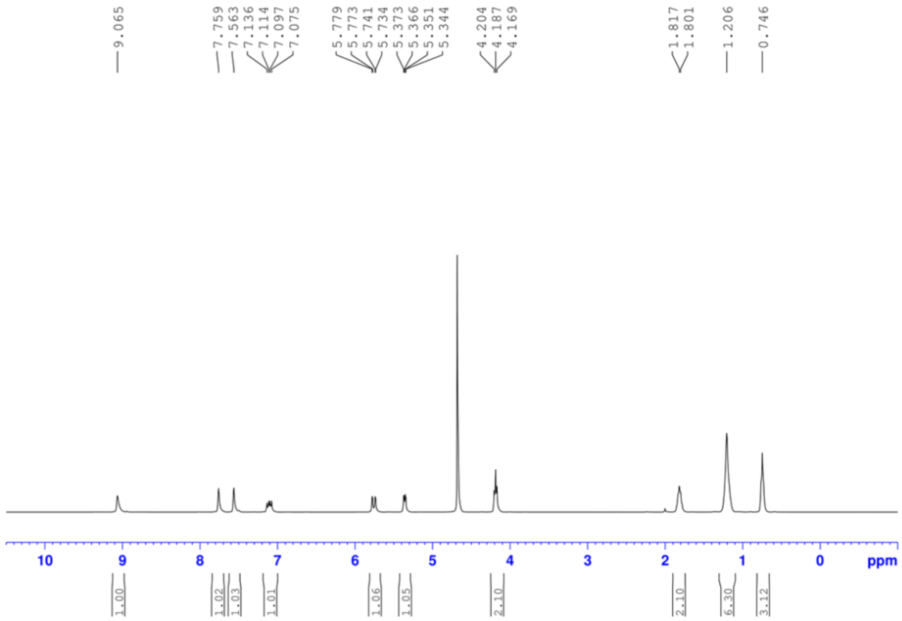

Figure S9: The proton NMR spectrum of IMC8 in D_2O.

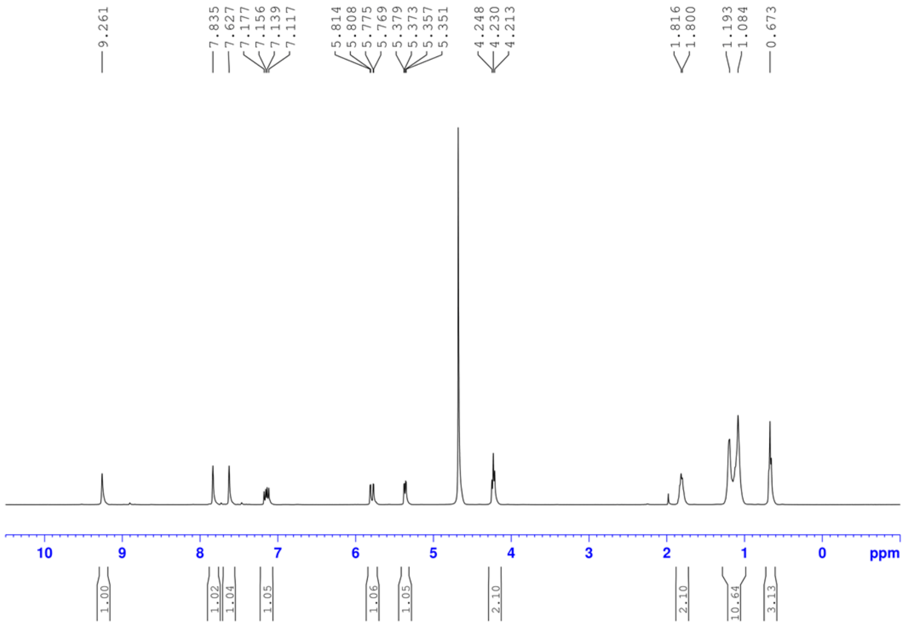

Hydrogel swelling experiment

A lyophilized gel is used to characterize the swelling ratio. The dry samples were incubated in deionized water until the hydrogel achieved an equilibrium swelling state in sealed containers at room temperature for five days [S1]. Remove the water on the surface of swollen hydrogels before weighting. The swelling ratio (SR) was calculated as follows:

$$SR = (W_s - W_d)/W_d$$

where W_s and W_d are the weights of swollen and dried hydrogels, respectively.

References:

S1. Paciello, A.; Santonicola, M. G. Supramolecular polycationic hydrogels with high swelling capacity prepared by partial methacrylation of polyethyleneimine. *RSC Adv.* **2015**, *5*, 88866-88875.
